# The Ubiquitin System and Jasmonate Signaling

**DOI:** 10.3390/plants5010006

**Published:** 2016-01-09

**Authors:** Astrid Nagels Durand, Laurens Pauwels, Alain Goossens

**Affiliations:** 1 Department of Plant Systems Biology, Vlaams Instituut voor Biotechnologie, B-9052 Ghent, Belgium; astrid.nagelsdurand@gmail.com (A.N.D); lapau@psb.vib-ugent.be (L.P.); 2 Department of Plant Biotechnology and Bioinformatics, Ghent University, B-9052 Ghent, Belgium

**Keywords:** ubiquitin, COI1, jasmonic acid, E3 ligase, JAZ, MYC2

## Abstract

The ubiquitin (Ub) system is involved in most, if not all, biological processes in eukaryotes. The major specificity determinants of this system are the E3 ligases, which bind and ubiquitinate specific sets of proteins and are thereby responsible for target recruitment to the proteasome or other cellular processing machineries. The Ub system contributes to the regulation of the production, perception and signal transduction of plant hormones. Jasmonic acid (JA) and its derivatives, known as jasmonates (JAs), act as signaling compounds regulating plant development and plant responses to various biotic and abiotic stress conditions. We provide here an overview of the current understanding of the Ub system involved in JA signaling.

## 1. Introduction to Jasmonates

Jasmonic acid (JA) together with its precursors and derivatives, referred to in general as jasmonates (JAs), play diverse roles in nearly all plant cells and organs. JAs regulate the response to wounding [1,2,3], to a large part by inducing specialized metabolism [4]. Likewise, resistance to necrotrophic pathogens is regulated mainly by JA, as well as by ethylene, whereas the response to biotrophic pathogens is regulated largely by salicylic acid (SA) [2,3,5]. JAs further contribute to plant plasticity by regulating responses to abiotic stresses such as temperature, drought and salt stress, thereby improving plant growth and productivity under adverse growth conditions [2,3]. Finally, JAs are also important in the regulation of plant growth, responses to light and general development [1,2,6]. The wide range of processes influenced by JAs is reflected in a marked alteration of the plant transcriptome upon treatment with JAs [7].

The production of JAs is triggered by wounding, caused mechanically or by herbivore attack, by infection with necrotrophic pathogens or by exposure to several abiotic stress conditions, among others, and supports the establishment of adequate defense responses against these threats [1,2]. JAs are cyclic oxylipins derived from fatty acid catabolism through the octadecanoid pathway [8]. Membrane damage causes release of α-linolenic acid, which is used as a substrate for JA biosynthesis. The first part of JA production takes place in the chloroplast, whereas the last step occurs in the peroxisome [1]. JA is then further metabolized into various derivatives, including the volatile compound methyl jasmonate (MeJA) [2,9]. Another JA derivative is formed by conjugation of JA with the amino acid Ile by JASMONATE RESISTANT1 (JAR1) [10,11]. JA-Ile has two chiral centers and the isomer (+)-7-iso-Jasmonyl-L-isoleucine has been identified as the endogenous bioactive form of the hormone [12]. JA-Ile perception in plants is guaranteed by a co-receptor complex in which an E3 ubiquitin (Ub) ligase is a critical component [13], putting an ubiquitination event central in the JA response. In this review, we will therefore focus on the multi-layered involvement of the Ub system in JA signaling.

## 2. Introduction to the Ub System

### 2.1. The Ub Conjugation Pathway: E1-E2-E3

Ubiquitin is a polypeptide (76 amino acids) present in all eukaryotes. Its protein sequence is highly conserved and plant Ub differs from human Ub by only three amino acids [14]. Ub and Ub-like polypeptides have a characteristic fold conferring tight packing and high structural stability [15].

**Figure 1:**
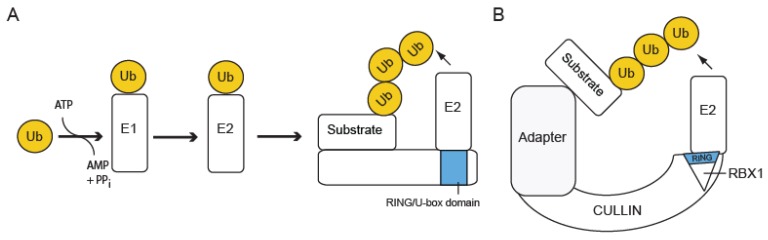
The Ub conjugation pathway. (**A**) Ubiquitin (Ub) is first activated and bound by E1 in an ATP-dependent reaction, after which it is transferred to E2. Then, E3 interacts simultaneously with the substrate and the E2-Ub conjugate, facilitating transfer of Ub to the substrate. E3 depicted in (A) is a single subunit which interacts via its RING or U-box domain with E2. An example of a RING-type E3 in JA signaling is DAD1-ACTIVATING FACTOR (DAF) [17]. The HECT-type E3s are not shown. (**B**) E3 can also occur as a multi-subunit E3 consisting of a CULLIN protein serving as scaffold for an adaptor module that binds the substrate, and the RING domain-containing RBX1 protein that binds E2. An example of a CULLIN-RING ubiquitin ligase (CRL) in JA signaling is SCF^COI1^.

At least three enzymes are required for Ub conjugation to a target protein (Figure 1A). First, Ub is activated by the Ub-activating enzyme (E1). This step requires ATP hydrolysis and yields a complex (E1-Ub) in which Ub is covalently bound to the E1 active-site Cys residue through a thioester bond. The Arabidopsis genome encodes two E1s (*UBA1-2*) [16].

The second step in the Ub pathway involves an Ub-conjugating enzyme (E2), during which Ub is transferred from E1-Ub to E2, where it is again covalently bound to the active-site Cys residue through a thioester bond. Arabidopsis encodes 48 proteins with a typical Ub-conjugating (UBC) domain, of which eight lack the active-site Cys and three are involved in conjugation of Ub-like proteins [18].

Finally, the Ub ligase (E3) forms a complex with E2-Ub and the substrate, facilitating transfer of Ub to the substrate, where it is covalently bound to a Lys residue. In most cases, E2-Ub and E3 interact non-covalently, although exceptions in which Ub is first covalently bound to the E3 through a thioester bond also occur. Because the E3 ligase is responsible for substrate recognition, it constitutes the main specificity determinant of the Ub system. More than 1,500 E3s are encoded in the Arabidopsis genome [19].

### 2.2. Classes of Ub Ligases (E3s)

Ub ligases can be subdivided into two structural types depending on their functionality as single-subunit proteins or multi-subunit complexes, and are further characterized into three mechanistic types that in most cases correlate with the presence of the conserved domain that mediates interaction with the E2-Ub (Figure 1). Single-subunit E3 ligases include proteins containing a conserved REALLY INTERESTING NEW GENE (RING) domain, a U-box domain or a Homologous to the E6-AP Carboxyl Terminus (HECT) domain. Whereas RING-type and U-box-type E3 ligases do not interact covalently with the E2-bound Ub, HECT-type E3s form a thioester-bound E3-Ub intermediary before Ub-transfer to the target protein [19,20,21]. The RING family of E3 ligases is characterized by the presence of a conserved RING domain, which mediates interaction with the E2-Ub. The RING domain contains eight conserved His or Cys metal binding residues that coordinate two Zn atoms forming a cross-brace secondary structure [21]. The model plant Arabidopsis encodes about 490 proteins that contain a RING domain [20].

To mediate interaction with the substrate, single-subunit E3 ligases often contain additional protein-protein interaction domains besides the HECT/RING/U-box domain. Alternatively, target recognition may be carried out by a separate subunit within a multi-subunit E3 complex. Complexes containing a RING domain, a CULLIN (CUL) protein and one or more adaptor modules function as complex RING-type E3 ligases and are known as CRLs (CUL-RING ligases, Figure 1B). The structural organization of CRLs is highly conserved between plants and animals: a CUL isoform functions as a scaffold accommodating the RING domain-containing protein RBX1a (RING BOX1) and a substrate-interacting module [19].

The most abundant type of CRL in plants are SKP1/CUL/F-box (SCF) CRLs characterized by the presence of CUL1 and a substrate-recognition module composed of ASK1/2 (ARABIDOPSIS SKP1 HOMOLOG 1/2) in combination with an F-box protein. In contrast to the situation in yeast or humans where a low number of F-box proteins is present (20 and 69, respectively), the Arabidopsis F-box family is very large and contains at least 700 members [19].

### 2.3. Versatility in the Ubiquitin System

Versatility in the Ub system is achieved by the introduction of flexibility as well as specificity at the level of both Ub conjugation and removal. During Ub conjugation, a covalent bond is formed between the C-terminal Gly residue of Ub and the ε-amino group of a Lys residue in the target protein, termed an isopeptide bond. The attachment of one Ub entity is referred to as mono-ubiquitination. Additional Lys residues within a single target protein can be modified with Ub resulting in multi-mono-ubiquitination. Reversibly, Ub-specific proteases known as deubiquitinases (DUBs) can recognize the C-terminal end of Ub and cleave specifically after Ub’s amino acid 76 [20], thereby recycling free Ub entities or restoring the unmodified function of the target protein. Reversible mono-ubiquitination of histones H2A and H2B has been associated with transcriptional repression or activation, respectively [22].

Ub contains seven Lys residues (K6, K11, K27, K29, K33, K48 and K63) that can be modified with Ub themselves, giving rise to the formation of Ub chains with seven different possible “linkages” [23]. Modification of a protein by covalent attachment of Ub chains is known as poly-ubiquitination. In addition, Ub can also form chains by attachment to the N-terminal amino group of the previous Ub moiety, forming linear Ub chains. The Ub-linkage within the chain leads to structural differences, which are in turn thought to be the basis for the different roles of the different linkage types of poly-Ub [23]. A final factor that further increases the complexity of the Ub system is the formation of mixed linkage chains and branched poly-Ub chains on target proteins, of which the role is still not well understood. The different signals arising by various structurally different forms of ubiquitination can be recognized and interpreted by proteins containing an array of ubiquitin-binding domains. In summary, cellular proteins can be modified by various and diverse ubiquitin signals, which alter protein activity, localization or fate. Thereby, Ub acts as a signaling component that can trigger numerous molecular events in cells [23].

Although much effort has been invested to identify motifs for Ub addition, identification of such a consensus sequence has failed, indicating that a universal Ub-motif does not exist. Instead, the mechanism of E3 ligases recruiting E2-Ub and the target protein, results in the creation of a “hot zone” in the target protein that is in close proximity to the Ub moiety [24,25,26]. While Ub proteomic studies have succeeded in detecting all eight possible Ub-linkage types in yeast and humans [24,25], K27-linked Ub chains and linear Ub chains have not been detected in plants [26]. The best characterized and most abundant linkage type arises from K48-linked Ub chains. Modification of a target protein with K48-linked poly-Ub marks the protein for proteolytic degradation [27]. The Ub system therefore plays a crucial role in regulated protein turnover. The second most abundant linkage type of poly-Ub identified on target proteins is K63-linked Ub chains. In yeast and mammalian cells, K63 poly-Ub has been associated with regulatory non-proteolytic functions such as DNA repair and kinase activation, and particularly with intracellular trafficking events. Although K63-linkage is poorly understood in plants, it has been implicated in intracellular trafficking by mediating endocytosis and subsequent vacuolar targeting of the auxin efflux carrier protein PIN2 [28]. K63-linked Ub chains have also been shown to be involved in plant iron homeostasis [29].

### 2.4. The 26S Proteasome

In all organisms studied so far, it has been shown that proteins that are poly-ubiquitinated with K48-linked Ubs are eventually degraded by a 2-MDa proteolytic complex: the 26S proteasome. This ATP-dependent multi-subunit protease is similarly organized in yeast, mammals and plants and can be divided in two particles: the 20S core particle and the 19S regulatory particle. The 20S core particle confers the three conserved proteolytic activities of the proteasome: peptidyl-glutamyl peptide-hydrolyzing (PGPH)-like, trypsin-like and chymotrypsin-like activity [30]. Each end of the 20S core particle is capped by a 19S regulatory particle, contributing to the recognition of poly-ubiquitinated proteins, their unfolding and the release of their poly-Ub chains [31].

### 2.5. Ubiquitin-Like Modifiers

The characteristic structure of Ub consists of a core β-grasp fold where a five-stranded β sheet appears to grasp a diagonally held α helix and is referred to as the Ub-fold. Several polypeptides have been identified that, despite their low sequence homology, show high structural similarity to Ub and share the common Ub-fold. In plants, these Ub-like modifiers (UBLs) currently include Nedd8 or RUB (related to Ub), SUMO (small Ub-like modifier), URM1 (Ub-related modifier-1), ATG8/12 (autophagy 8/12), MUB (membrane anchored Ub), UFM1 (Ub-fold modifier-1) and HUB1 (homology to Ub-1). Like in the case of Ub, UBL conjugation generally depends on an E1-E2-E3 cascade and targets the ε-amino acid group of Lys residues [32]. Although UBLs share a similar fold, their functions and properties differ from Ub and from each other.

Among the UBLs, Nedd8 is most closely related to Ub and is strongly conserved across species [32]. Three copies of Nedd8 are encoded in the Arabidopsis genome (*RUB1-3*), and neddylation is essential for plant growth and development [33]. In Arabidopsis, ECR1 and AXR1 or AXL form a heterodimeric Nedd8-activating enzyme (E1, [34,35]) while *RCE1* and *RCE2* (for RUB-CONJUGATING ENZYME) encode two Nedd8-specific conjugating enzymes (E2s, [36]).

The best characterized Nedd8-conjugated proteins in all eukaryotic organisms are CULs, the scaffold proteins of CRLs. Remarkably, the RING component of CRLs, RBX1, was found to be necessary, not only for the ubiquitination of CRL targets but also as a Nedd8 E3 ligase to catalyze CUL neddylation [37,38]. Neddylation of CUL is essential for the activation of CRL complexes and is crucial for regulating their activity [19]. In mammals and yeast, several other RING-type E3 ligases have been reported to function in Nedd8 conjugation, in addition to RBX1 [39]. Finally, similar to DUBs which make ubiquitination a reversible modification, DENEDDYLASE1 (DEN1) and the COP9 signalosome (CSN) have Nedd8-protease activity [40,41]. CSN is an evolutionary conserved multi-protein complex that promotes CUL deneddylation, which is essential for the correct regulation and activity of CRL-type E3 ligases [42]. Until recently, only two non-CUL neddylation substrates were known in plants: DDB1, a CUL4-CRL subunit, and ML3, a protein involved in pathogen responses whose exact function is not well understood [40]. The accumulation of a broad range of Nedd8-conjugates in Arabidopsis *den1* mutants, however, indicates that several other neddylation targets exist besides CULs, DDB1 and ML3 [41].

The SUMO pathway is also essential for plants because mutations cause embryo lethality. The SUMO conjugation cascade in plants involves a heterodimeric E1 (SAE1a/b and SAE2, for SUMO ACTIVATING ENZYME) and the SUMO-conjugating enzyme SCE1 (E2). At least two SUMO-specific E3 ligases are known in plants, SIZ1 and HYP2/MMS21 [43,44]. In addition, SUMO can also form chains, generating poly-SUMOylated proteins. This modification does not serve as a signal for proteolytic degradation by the proteasome but rather seems involved in modulating the ability of substrates to interact with other proteins [45]. In plants, various forms of abiotic stress cause a dramatic rise of SUMO conjugates, mainly in the nucleus [43,46,47]. Accordingly, in yeast and mammals, SUMO modification has been shown to activate transcription factors (TFs) or to cause translocation of cytosolic factors to the nucleus [45]. SUMOylation is therefore thought to help shift the plant from growth to a protective mode [43].

Little is known about the function or conjugation of UFM1, MUB, HUB1 and ATG8/12 in plants. While knowledge of UFM1 is completely missing in plants, all E1, E2 and E3 enzymes for UFM1 conjugation have already been characterized in mammals [48,49]. MUB, a UBL present in animals, fungi and plants, is anchored to the membrane owing to the presence of a prenylation signal at its C-terminus instead of the usual di-Gly motif [50]. In yeast and mammals, HUB1 functions non-covalently. Accordingly, no E1-E2-E3 cascade has been identified for this UBL. Finally, ATG8 and ATG12 are UBLs with an essential role in autophagy that is conserved in yeast, humans and plants [51,52,53].

## 3. Mechanism and Importance of Ubiquitination of JAZ Proteins in JA Signaling

**Figure 2:**
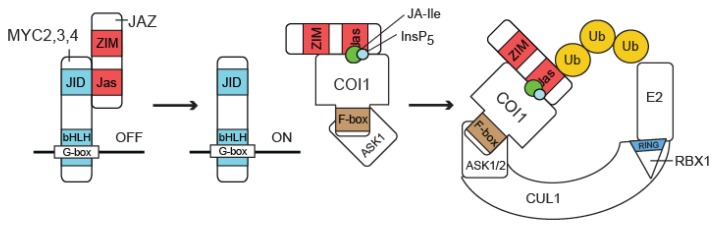
Overview of the core JA-signaling pathway. In the absence of JA-Ile, the activity of the TFs MYC2/3/4, bound to the G-box of JA-responsive promotors, is repressed by interaction with a JAZ protein that binds to the JAZ-interacting domain (JID) of MYC2/3/4 through their Jas domain. Upon perception of the hormone (green dot) by the co-receptor complex composed of a JAZ protein and the F-box component COI1 (SCF^COI1^), which is potentiated by inositol pentakisphosphate (InsP5, blue dot), the CUL-RING E3 ligase (CRL) binds with SCF^COI1^ and ubiquitinates the JAZ protein. Subsequently, JAZ proteins are degraded and MYC2/3/4 activate transcription of JA-responsive genes eventually resulting in a JA response. Figure adapted from Pauwels and Goossens [60].

The core JA-signaling module is primarily defined as being composed of: (i) MYC2, a key TF regulating the expression of JA-responsive genes [54], (ii) Jasmonate ZIM-domain (JAZ) proteins, repressors that inhibit MYC2 activity in the absence of the hormone [55,56] and (iii) CORONATINE INSENSITIVE 1 (COI1) that acts as the JA receptor *in vivo* and, in response to JA-Ile, targets the JAZ repressors for proteolytic degradation [12,57] (Figure 2). The co-repressors Novel Interactor of JAZ (NINJA) and TOPLESS (TPL) are closely connected to the core JA-signaling complex, because they mediate the repressor-effect of JAZ proteins [58]. The components of the core JA-signaling module are considered essential for JA-signal transduction, because mutations affecting this complex disturb many (or all) JA responses [1,9,59]. The effect of ubiquitination by or of COI1, JAZ and MYC proteins and the importance thereof in JA signaling will be discussed in the following sections.

### 3.1. COI1 Links the Ubiquitin System to JA Signaling

Coronatine (COR) is a phytotoxin produced by *Pseudomonas syringae* that structurally and functionally resembles JA-Ile. Based on the property of COR and JAs to inhibit seedling root and shoot growth, a screen for COR-insensitive mutants led to the discovery of the *CORONATINE-INSENSITIVE1* (*COI1*) gene. Loss-of-function *coi1-1* mutants are insensitive to COR/JA and are male sterile in Arabidopsis [61]. In addition, *coi1-1* mutants are resistant to *P. syringae* infection [5,62,63]. Because COR is a molecular mimic of JA-Ile, it is perceived by the same receptor and can trigger JA signaling, which antagonizes SA-dependent defense mechanisms that are normally needed to limit *P. syringae* growth. This explains why *coi1-1* plants are resistant to *P. syringae* [1,5].

The COI1 protein was characterized as an F-box protein that, besides its N-terminal F-box domain, also contains 16 leucine rich repeats (LRRs) [64]. The F-box domain of COI1 is characteristic for proteins that associate with SCF Ub ligase complexes and the protein is closely related to other F-box proteins with a function in hormone signaling. COI1 has been shown to associate with RBX1, CUL1, and either ASK1 or ASK2 to assemble functional SCF^COI1^ complexes in Arabidopsis [65,66,67]. Accordingly, plants deficient in components or regulators of SCF complexes also show impaired JA responses [54,59,68,69].

**Figure 3:**
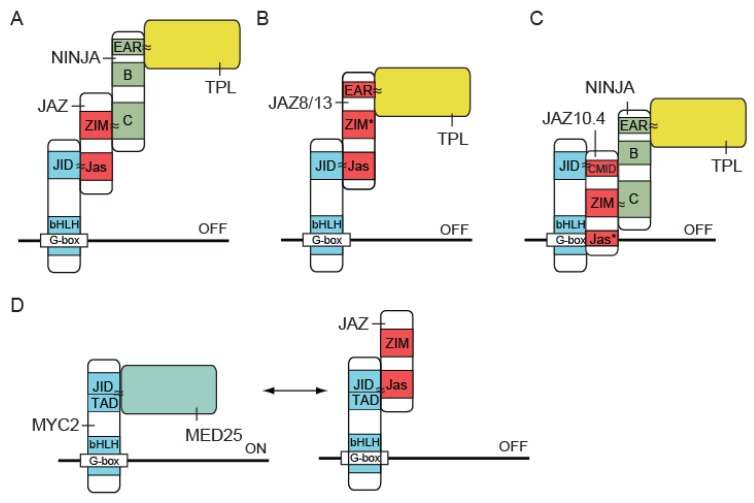
JAZ proteins repress JA signaling. (**A**) JAZ proteins interact with bHLH-type TFs, such as MYC2/3/4, mediated by direct binding between the Jas and JAZ-interacting domain (JID) domains, respectively. The ZIM domain of most JAZ proteins can interact with the adapter protein Novel Interactor of JAZ (NINJA). Through an ERF-associated amphiphilic repression (EAR) motif, NINJA is capable of recruiting the co-repressor TOPLESS (TPL) to the complex. (**B**) The ZIM domains of some JAZ proteins, such as JAZ8 and JAZ13, are unable to bind NINJA, but these JAZ proteins contain an EAR motif themselves. (**C**) Alternative splicing of certain *JAZ* transcripts leads to truncated JAZ proteins lacking part of the Jas domain. In the case of JAZ10.4, an N-terminal cryptic MYC2-binding domain (CMID) mediates interaction with bHLH-type TFs such as MYC2/3/4. Both JAZ8/13 and JAZ10.4 have deviating Jas domains and do not associate with SCF^COI1^ in a JA-Ile dependent way. (**D**) The interaction of JAZ proteins with MYC2 is mediated by both the JID and the transcriptional activation domain (TAD). This interaction competes with the binding of this surface with the co-activating mediator subunit MED25. Figure partially adapted from Pauwels and Goossens [60].

In 2010, Sheard, *et al.* [70] published the crystal structure of COI1 in complex with ASK1, JA-Ile and the JAZ1 degron, the region of JAZ1 necessary to mediate its SCF^COI1^-dependent degradation. This finally revealed the exact mechanism for JA perception. The overall structure of COI1 resembles that of the auxin receptor F-box protein TRANSPORT INHIBITOR RESPONSE 1 (TIR1). The 18 tandem LRRs of COI1 form a horseshoe-shaped solenoid domain, housing the JA-Ile binding pocket. High-affinity JA-Ile binding, however, requires both COI1 and the JAZ degron, thus implying JA perception is mediated by a co-receptor complex consisting of COI1 and JAZ. Part of the JAZ1 degron interacts with JA-Ile, while the other part interacts with COI1. The JAZ1 degron is therefore referred to as a bi-partite degron. Four loops (loop-2,-12,-14 and loop-C) protrude at the top surface of the COI1 LRR domain and are important for JA-Ile and JAZ binding. Finally, inositol-pentakisphosphate was identified as a COI1 cofactor, binding at the center of the protein, underneath the JA-Ile binding pocket, and shown to be crucial for the formation of a high-affinity co-receptor complex [70]. Since the establishment of the general mechanism of JA-Ile perception by COI1-JAZ, two main exceptions to the rule have been recognized. First, a JAZ10 splice variant (JAZ10.4) was found to lack the Jas domain, comprising the JAZ degron. JAZ10.4 is therefore unable to interact directly with COI1, independent of the presence or absence of the hormone and is thus resistant to JA-induced degradation [70,71]. Second, JAZ8 is a JAZ protein lacking a conserved motif within the JA-Ile binding region of the model bi-partite JAZ degron, is therefore unable to strongly associate with COI1 in the presence of JA-Ile [72]. Since both JAZ10.4 and JAZ8 maintain the ability to interact with MYC2, both can repress JA responses in the presence of the hormone (Figure 3) [72]. Sequence variation within the JAZ degron motif may impose the requirement of a minimal JA-Ile concentration in order to turn on the JA response or may provide a mechanism to regulate the strength of the JA response once initiated.

The presence of ASK1 in complex with COI1 during crystallization was later found to be essential, because COI1 turned out to be an unstable protein when dissociated from the SCF complex. COI1 instability is, again, mediated by the Ub system, because its degradation was proteasome dependent and involved ubiquitination at Lys297 [73]. COI1 protein levels are thus strictly regulated and maintained at a level essential for proper activation of JA responses.

SCF^COI1^, thus, links the Ub system to JA perception by forming a co-receptor complex with JAZ for the bioactive JA-Ile, as well as for its molecular mimic COR. Perception of the phytohormone by the F-box protein COI1 leads to ubiquitination of JAZ repressors by SCF^COI1^ and their subsequent degradation by the proteasome. This causes de-repression of TFs involved in the activation of JA-mediated responses. The variety of phenotypes associated with the *coi1-1* mutation, however, suggests that SCF^COI1^ might have multiple targets [65]. In addition, because the *jar1* mutant, defect in the enzyme responsible for JA-Ile production, does not display all the defects that are observed in the *coi1* mutant [74], other signals than JA-Ile might be recognized by the same or a different receptor to activate JA signaling. Conversely, it cannot be excluded that redundancy in JA-Ile-conjugating enzymes may exist.

### 3.2. JAZ Proteins Repress JA Signaling

Jasmonate ZIM-domain (JAZ) proteins have been shown to constitute the missing link between the transcriptional regulation of JA responses and the perception of JA. In the absence of JAs, members of the JAZ protein family repress expression of JA-responsive genes by inhibiting MYC2 activity. Upon treatment with the hormone, JAZ proteins are degraded by the 26S proteasome, thereby allowing transcriptional activation of JA-responsive genes. This degradation is dependent on direct interaction between JAZ and COI1 [55,56,70].

The JAZ protein family has 13 members in Arabidopsis [55,75] and homologs have been identified in several plant species, both dicots and monocots, but not outside the plant kingdom [55,75,76]. JAZ proteins contain three conserved domains: the zinc-fingers expressed in the inflorescence meristem (ZIM) domain, a region of weak homology at the N-terminus and the C-terminal Jas domain which is most strongly conserved. The Jas domain is essential for JAZ stability, because it constitutes the interaction platform between JAZ and COI1 upon hormone treatment (Figure 2) [55,56,77]. Mutation of essential amino acid residues within the Jas domain generates plants with a JA-insensitive phenotype similar to that of *coi1-1* [77]. The Jas domain is also essential for the interaction with several TFs such as MYC2/3/4 (Figure 3A) [55,56,60,77,78]. Mutant or “divergent” JAZ proteins, such as JAZ10.4 and JAZ8, maintain the ability to interact with MYC2 because they have a second, N-terminal cryptic MYC2 binding domain (CMID) or a deviating Jas domain, respectively, that enables specific binding to MYC2 but not association with SCF^COI1^ (Figure 3B,C) [70,71,72].

The ZIM domain refers to a 36-amino acid domain, containing a conserved motif, shared by ZIM and ZIM-like (ZML) proteins. Within the ZIM domain, a strongly conserved amino acid pattern forms the TIFY motif [79]. The ZIM domain mediates hormone-independent homo- and hetero-dimerization of JAZ proteins [71,80] as well as interaction with NINJA [58] (Figure 3A). The newest member of the JAZ family, JAZ13, contains a divergent TIFY motif and Jas domain (Figure 3B). This protein was shown to be a functional member of the JAZ family and to repress JA responses through interaction with the TF MYC2. However, the divergent TIFY motif does not mediate hetero-dimerization of JAZ13 with other members of the JAZ-family [75]. JAZ13 is most closely related to JAZ8, and both proteins contain a divergent Ethylene Response Factor (ERF)-associated amphiphilic repression (EAR) motif [72,75]. As mentioned earlier, the repressor function of JAZ proteins is generally mediated by interaction with NINJA, which recruits the co-repressor TPL through interaction with its EAR motif [58]. Accordingly, while JAZ8 and JAZ13 are unable to bind NINJA, the presence of an EAR motif mediates direct interaction with the co-repressor TPL [72,75] (Figure 3B).

Direct proof of the *in vivo* ubiquitination of JAZ proteins is, however, still scarce. To our knowledge, only two family members have been found to be modified with Ub or a UBL *in vivo*, *i.e.*, JAZ6 and JAZ12 [67,81]. Evidence for the *in vivo* ubiquitination of these two JAZ proteins was obtained through proteome-wide or targeted mass spectrometry-based approaches that led to the identification of a di-glycine modification of the proteins; a modification that is derived from Ub or a UBL and remains on the target protein after trypsin digestion, an intermediary step during the preparation of samples for mass spectrometric analysis [67,81].

### 3.3. MYC2 Regulates the Transcription of JA-Responsive Genes

The TF MYC2 plays a central role in regulating the transcriptional reprogramming in response to JAs. *MYC2* was first identified as *JASMONATE INSENSITIVE-1* (*JIN1/JAI1*) in two different screens for mutants with a reduced JA sensitivity. The *myc2* mutant shows reduced root growth inhibition and anthocyanin production when treated with MeJA compared to wild-type plants. *MYC2* expression is induced by JAs or wounding, and overexpression (OE) of the TF renders plants hypersensitive to JAs [54,82].

MYC2 encodes a nuclear localized basic helix-loop-helix-leucine zipper (bHLH)-type TF that specifically regulates numerous branches of the JA response, including the wounding response, defense against herbivores and pathogens, elicitation of specialized metabolism, oxidative stress tolerance, drought and other abiotic stress responses and regulation of degreening in senescence [4,83,84].

Through its basic amino acids, MYC2 binds to the G-box, a CACGTG palindrome hexamer, and G-box related motifs present in the promoters of a great number of genes that are activated by JA [85,86,87]. The MYC2 promoter itself also contains a G-box motif and the TF regulates its own transcription [86]. Because most *JAZ* genes also have a G-box (or a variant of it) in their promoter, a negative feedback loop is induced during JA signaling. JA-induced MYC2-mediated induction of *JAZ* expression therefore results in a pulsed response followed by subsequent desensitization, avoiding a harmful runaway response [55,56].

The bHLH domain is required for the formation of homo- or hetero-dimers with other related TFs [78]. The N-terminal part of MYC2 contains a transcriptional activation domain (TAD), that mediates interaction with the Mediator complex subunit MED25 for transcription initiation [78,88,89] (Figure 3D), and a JAZ interaction domain (JID) that mediates interaction with the JAZ repressors [55,78].

The transcriptional reprogramming induced by JAs has been shown to enclose two different transcriptional waves. An early transcriptional wave induces expression of genes encoding primary regulators of JA signaling, such as the JAZ proteins and MYC2. A subsequent wave consists of both positive and negative regulation of genes including other TFs [7]. *Myc2* mutants are not fully impaired in all JA-mediated responses. For that reason, the transcriptional reprogramming following JA perception cannot be entirely performed by MYC2 [59]. Indeed, the TFs MYC3/ATR2 and MYC4 that are closely related to MYC2 were also shown to activate JA-dependent transcription upon perception of the hormone [78,90]. Similarly to MYC2, MYC3/4 transcriptional activity was directly inhibited by binding with JAZ proteins and loss-of-function mutations in any of the two TFs rendered the plants partially insensitive to JA and aggravated the JA-insensitive phenotype when combined with the *myc2* mutant [78]. Recently, Gasperini and colleagues demonstrated that the expression of *MYC2/3/4* in the root meristem is cell-layer specific and mutually exclusive in the case of MYC3 and MYC4 [91].

The structural basis of JAZ repression of MYC TFs was recently elucidated using the interaction between MYC3 and JAZ9 as a model [92]. The Jas domain of JAZ repressors is required for both the formation of the COI1-JA-Ile-JAZ co-receptor complex and the interaction with the JID domain of MYC TFs. Through crystallographic assays, it has been shown that the Jas motif undergoes a structural switch, from a partially unwound helix when bound to the hormone, to a complete α-helix when bound to MYC3. This α-helix competitively inhibits MYC3 interaction with MED25, thereby preventing the transcriptional activation of JA-responsive genes, which is normally exerted through the association with the Mediator complex [92]. Accordingly, mutations of conserved residues within the JID domain of MYC TFs affecting the JAZ-MYC interaction were shown to cause constitutive activation of JA-responsive genes in the absence of hormone treatment [90,91].

## 4. Ubiquitination in JA Signaling beyond the Core Module

In addition to the physical interaction between them, all known JAZ and MYC2/3/4 proteins specifically interact, physically or genetically with several other proteins [60,83,93]. Many of these JAZ- and MYC-interacting proteins form points of crosstalk with other signaling pathways, further fine-tuning the JA response. Here, we will list and discuss those that involve a link with ubiquitination processes. In addition, we will also discuss components of the Ub system that affect the JA response, but whose physical or genetic link to the pathway is still not well understood. An overview is given in Table 1.

**Table 1:** Overview of Ub-system components with a role in jasmonic acid (JA) signaling.

Protein or Protein Complex	Ub-System Class	Candidate Target	Target Type	Evidence	Process	Reference
SCF^COI1^	Ub E3: CRL	JAZ	repressor	Binding, stability, genetic	JA signaling	[55,56]
DAF	Ub E3: RING	DAD1	enzyme	Hypothetical	JA biosynthesis	[17,94]
KEG	Ub E3:	ABI5	TF	Binding, stability, *in vitro* assay, genetic	ABA signaling, JA signaling	[67,95,96,97]
	RING	ABF1	TF			
		ABF3	TF			
RGLG3 RGLG4	Ub E3: RING	-	-	-	JA signaling, SA-JA crosstalk	[98,99]
PUB10	Ub E3: U-box	MYC2	TF	*In vitro* assay	JA signaling	[100]
PUB10/11	Ub E3: U-box	MYC3 MYC4	TF	Binding, stability	JA signaling	[100]
BOI	Ub E3: RING	BOS1/MYB108	TF	Binding, stability, *in vitro* assay	Cell death JA signaling	[101,102,103]
MtMKB1	Ub E3: RING	MtHMGR	enzyme	Binding, stability	ERAD JA signaling	[104]
OsHOS1	Ub E3: RING	OsEREBP1 OsEREBP2	TFs		JA signaling, mechanosensing	[105]
At/OsHOS1	Ub E3: RING	At/OsICE1	TF		JA signaling, abiotic stress responses	[106,107]
-	-	JAV1	-	Stability	JA signaling	[108]
-	-	ORA59	TF	Stability	SA-JA crosstalk	[109]
TIC	-	MYC2	TF	Binding, stability	JA signaling, circadian rhythm	[110]
UBP12 UBP13	DUBs	TIFY8	Transcrip-tional regulator	Binding	biotic stress responses, photoperiodicity	[111,112,113]
AXR1/AXL /ECR1	Nedd8 E1	CUL	CRL component	Genetic, stability	CRL activity regulation, JA signaling	[34,35,114]
RCE1/2	Nedd8 E2	CUL	CRL component	Genetic	CRL activity regulation, JA signaling	[36,115]
CSN	deneddylase	CUL	CRL component	Genetic	CRL activity regulation, JA signaling	[116]
SIZ1	SUMO E3	multiple	multiple	Genetic	Pleiotropic, JA signaling	[117]

-, unknown; stability: protein stability is altered e.g., in mutant or upon proteasomal inhibition; binding: protein-protein interaction either direct or indirect; *in vitro* assay: *in vitro* (poly)-ubiquitination of target using recombinant proteins; TF: transcription factor; CRL: CUL-RING ligase.

### 4.1. The Ub E3 Ligase DAF Is Involved in JA Biosynthesis

The phospholipase A1 protein DAD1 (DEFICIENT IN ANTHER DEHISCENCE1) catalyzes the initial step of JA biosynthesis, the release of α-linolenic acid from chloroplast membranes. *Dad1* mutants are deficient in anther dehiscence and in pollen maturation, eventually resulting in a male-sterile phenotype, which can be rescued by exogenous application of JA to the flower buds [94]. Similar phenotypes were observed when expression of the E3 Ub ligase *DAF*/*ATL73* (*DAD1-ACTIVATING FACTOR*) was suppressed [17]. DAF belongs to the ATL sub-group of RING-type E3 Ub ligases [118], linking the Ub system to JA biosynthesis in addition to JA signal transduction. The E3 ligase DAF acts upstream of DAD1 and is necessary for the expression of *DAD1*, to enable correct JA-mediated flower development. The function of DAF is dependent on the integrity of its RING domain, but no DAF ubiquitination target has been confirmed yet [17].

### 4.2. The Ub E3 Ligase KEG Interacts with JAZ12

*KEG* encodes a 178-kDa-large protein assembled from the combination of four different types of functional domains: an N-terminal RING domain, a serine/threonine protein kinase domain, nine consecutive ankyrin repeats and twelve consecutive HERC2-like repeats. Seedlings carrying loss-of-function mutations in *KEG* (*keg^KO^*) arrest growth soon after germination. This seedling-lethal phenotype is mediated, at least in part, by ABA hypersensitivity [97]. In the absence of ABA, KEG interacts with and ubiquitinates the TF ABSCISIC ACID INSENSITIVE5 (ABI5) in the cytosol, thereby mediating proteasome-dependent degradation of ABI5 [96,97]. Additionally, the ABI5-related TFs ABF1 and ABF3 were also shown to be ubiquitinated *in vitro* by KEG [95]. ABI5, ABF1 and ABF3 positively regulate ABA responses, including ABA-induced post-germinative growth arrest under adverse environmental conditions [95,96,97]. Nevertheless, restoration of ABA-sensitivity in *keg^KO^* seedlings does not complement all mutant phenotypes.

Recently, KEG has been reported to be a positive regulator of JAZ12 protein levels. Although JAZ12 behaves as a canonical JAZ protein with respect to the JA-signaling pathway (it represses MYC2 activity and it associates with SCF^COI1^ in the presence of JA, leading to its rapid degradation after treatment with JA), it has nonetheless specific features because KEG specifically interacts with JAZ12 and not with other members of the JAZ protein family. In addition, both ABA treatment and knockdown of *KEG* leads to a decrease in JAZ12 protein levels. Correspondingly, *KEG* overexpression is capable of partially inhibiting COI1-mediated JAZ12 degradation. These results provide additional evidence for KEG as an important factor in plant hormone signaling and a positive regulator of JAZ12 stability [67].

### 4.3. The Ub E3 Ligases RGLG3/4 Positively Regulate JA Signaling

*RGLG3* and *RGLG4* belong to the *RGLG* (*RING DOMAIN LIGASE*) family of E3 ligases that contains five members in Arabidopsis [21,99]. These E3 ligases have been reported to function redundantly as positive regulators of JA responses [99]. Furthermore, RGLG3 and RGLG4 are involved in the regulation of crosstalk between SA and JA in response to infection with the fungal pathogen *Fusarium moniliforme* [98]. The molecular mechanisms underlying RGLG3 and RGLG4 function in these processes and the ubiquitination target(s) of RGLG3 and RGLG4 remain to be identified.

### 4.4. The Ub E3 Ligase PUB10 Regulates MYC2 Protein Levels

Similar to the RING domain, the U-box domain also serves as the E2 docking site in U-box-type E3s (Figure 1A). However, contrary to the RING domain, the U-box domain does not bind Zn atoms but depends on a network of hydrogen bonds that is further stabilized by hydrophobic interactions and salt bridges [119]. Arabidopsis encodes 64 proteins that contain a U-box domain [20]. The PLANT U-BOX PROTEIN 10 (PUB10) belongs to the family of U-box-type E3 ligases and mediates the ubiquitination and subsequent degradation of the TF MYC2 in a proteasome-dependent manner [100]. In addition, both PUB10 and its closest homolog (PUB11) can interact with MYC3 and MYC4, though further ubiquitination of these MYC2-related TFs has not been investigated yet. Although MYC2 accumulation is increased in *pub10* loss-of-function plants, it remains an unstable protein, indicating that additional E3 ligases might function in regulating MYC2 protein levels *in planta* [100].

### 4.5. The Ub E3 Ligase BOI and Its Target BOS1 Link Cell Death to JA Signaling

BOTRYTIS SUSCEPTIBLE 1 INTERACTOR (BOI) is a RING-type E3 ligase that negatively regulates pathogen- and stress-induced cell death, thereby contributing to plant disease resistance and abiotic stress tolerance. In addition to a C-terminal RING domain, BOI contains a central domain that is essential for interaction with the MYB TF BOTRYTIS SUSCEPTIBLE1 (BOS1/MYB108) [101]. BOS1 was first identified in a screen for factors controlling JA responses in stamens, where it functions together with MYB24 and downstream of MYB21 to mediate stamen and pollen development in response to JAs [102]. BOS1 was later shown to be an ubiquitination target of BOI *in vitro*, and its *in vivo* protein levels are regulated by the 26S proteasome [101]. Like BOI, BOS1 has also been shown to be involved in plant resistance to pathogens and tolerance to abiotic stresses [103]. *BOS1* expression is induced by infection with necrotrophic pathogens in a COI1-dependent fashion, whereas JA treatment causes repression of *BOI*, implicating that the pathway leading to disease- or stress-induced cell death that is regulated by BOI-BOS1 is, at least partially, controlled by JA [101,103].

### 4.6. The Ub E3 Ligase MtMKB1 Links JA Signaling to Protein Quality Control

JA perception is known to trigger the production of specialized metabolites in plants [4]. In the model legume *Medicago truncatula*, JA induces the production of triterpene saponins and the E3 ligase MAKIBISHI1 (MtMKB1) has been shown to regulate the production of triterpene saponins in *M. truncatula* in response to JA treatment. MtMKB1 is a RING membrane-anchor (RMA)-like protein located in the endoplasmic reticulum (ER), like its mammalian counterpart RMA1 that is involved in the ER-associated degradation (ERAD) protein quality control system. In yeast and mammals, ERAD-mediated control of 3-HYDROXY-3-METHYLGLUTARYL-COA (HMGR) levels regulates the production of sterols. In accordance with the requirement of HMGR activity to supply precursor molecules for the production of triterpene saponins, JA signaling employs the regulation of MtHMGR by MtMKB1 to manage the production of bioactive defense compounds (triterpene saponins) in the legume *M. truncatula* [104].

The Arabidopsis genome encodes three genes with sequence homology to human *RMA*: *AtRMA1/2/3*. All three Arabidopsis homologs are located in the ER, and are active as Ub E3 ligases *in vitro* [120]. AtRMA2 has been reported to physically interact with and ubiquitinate AUXIN BINDING PROTEIN 1 (ABP1), leading to proteasome-dependent degradation of ubiquitinated ABP1 [121]. The biological significance of this interaction is, however, not clear, because ABP1 has recently been shown not to be essential for neither auxin signaling, nor general plant development [122]. A possible role for the Arabidopsis RMA-homologs in JA signaling remains to be investigated.

### 4.7. The Ub E3 Ligase HOS1 Links JA Signaling to Multiple Developmental and Abiotic Stress Responses

JAs further contribute to plant plasticity by regulating responses to abiotic stresses such as temperature stress or root curling in response to mechanical stimuli [123,124]. The Ub E3 ligase HIGH EXPRESSION OF OSMOTICALLY RESPONSIVE GENE1 (HOS1) has been involved in both processes. First, studies using rice as a monocot model plant have shown that the expression of the receptor-like kinase protein ROOT MEANDER CURLING (OsRMC) is induced by JA treatment and acts as a negative regulator of JA-mediated root curling [124]. The expression of OsRMC is regulated by two ERF TFs: OsEREBP1 and OsEREBP2. Accumulation of these TFs is modulated, in turn, by the rice Ub E3 ligase OsHOS1, which mediates the proteasome-dependent degradation of the TFs. In addition, knockdown of OsHOS1 leads to a reduced sensitivity to JA-induced root growth inhibition, confirming this E3 ligase is involved in the JA-signaling pathway in rice [105]. Second, tolerance to cold/freezing stress in Arabidopsis is mediated by the bHLH TFs ICE1 and ICE2. In the absence of JAs, expression of *ICE1* and *ICE2* is repressed by the JA-signaling machinery. Exposure to cold leads to elevated biosynthesis and accumulation of JAs in the plant, triggering *ICE1/2* expression [123,124]. Remarkably, ICE1 protein stability is also regulated through ubiquitination by HOS1, both in Arabidopsis and in rice [106,107].

### 4.8. JAV1 Protein Stability Is Regulated by COI1-Dependent JA Perception

The VQ-motif-containing protein JAV1 (JASMONATE-ASSOCIATED VQ MOTIF GENE 1) was identified as a negative regulator of JA-mediated plant defense responses against pathogens and herbivore insects. Notably, COI1-dependent JA perception leads to JAV1 protein degradation, which is proteasome dependent and thus regulated by the Ub system. Because SCF^COI1^ does not interact directly with JAV1, the E3 Ub ligase responsible for JAV1 ubiquitination and subsequent degradation remains to be identified [108].

### 4.9. Protein Stability of the Transcription Factor ORA59 Affects JA-SA Crosstalk

During JA signaling, MYC2 negatively regulates genes involved in pathogen defense, as well as positive regulators of these genes. Instead, expression of those genes in response to JA is dependent on two of these positive regulators: the TFs *ORA59* (*OCTADECANOID-RESPONSIVE ARABIDOPSIS AP2/ERF59)* and *ERF1* [83]. During SA-JA antagonism, the activation of the SA-signaling pathway induces ORA59 protein degradation, thus suppressing JA signaling through this TF. The mechanism by which SA signaling suppresses ORA59 accumulation remains unknown. However, ORA59 destabilization is independent of COI1 activity and is therefore not mediated by the interference of SA signaling with the ubiquitination of JAZ proteins by COI1 [109].

### 4.10. Light Signaling Alters JAZ Protein Stability through Gibberellic Acid (GA) Signaling

DELLA proteins, repressors of GA responses, interact with both MYC2 and JAZ proteins to modulate JA responses. These interactions are especially important during shade avoidance responses, when resource allocation is primarily directed from defense to growth [125]. At low GA concentrations, DELLAs compete with MYC2 for JAZ binding, modulating the JA response. When shade is perceived by the plant (low R:FR ratio), GA signaling triggers degradation of DELLAs, increasing the capacity of JAZ proteins to bind and inactivate MYC-type TFs. Moreover, low R:FR light conditions increase the stability of several JAZ proteins, while simultaneously increasing MYC2/3/4 turnover, resulting in increased repression of the JA response. In addition, one DELLA protein (RGL3) has been shown to positively regulate MYC2 and thus to be required for full activation of the JA responses (reviewed in [6,126]).

### 4.11. TIC Synchronizes JA Signaling to the Circadian Clock

Another aspect of light signaling that is integrated by JAs is circadian signaling [110,127,128]. Both *MYC2* expression and protein accumulation seem to be under control of the circadian clock [110]. This rhythmic MYC2 accumulation is regulated by the Ub system, because interaction of MYC2 with TIME FOR COFFEE (TIC), a component of the circadian clock, leads to proteasome-dependent degradation of MYC2. In addition, JA perception is also regulated in a circadian manner, because the expression of *COI1* is also controlled by TIC [110]. Finally, circadian JA accumulation has been shown to contribute to the plant’s defense mechanism against herbivore insects as well as fungal pathogens [127,128].

### 4.12. A Possible Role for Deubiquitination in JA Signaling

The ZIM domain of JAZ proteins mediates dimerization of JAZ proteins [71,80], as well as interaction with NINJA [58]. A strongly conserved amino acid pattern within the ZIM domain forms the TIFY motif [79]. TIFY8 constitutes an atypical member of the TIFY protein family, because it contains a TIFY motif but lacks the Jas domain characteristic of JAZ proteins [111]. Although no role for TIFY8 in JA responses has been demonstrated yet, TIFY8 was present in protein complexes that included UBP12 [111], an ubiquitin protease that was reported to be required together with its closest homolog (UBP13) for immunity against *Pseudomonas syringae* in tomato [113] and to act in circadian clock and photoperiodic flowering regulation [112], processes that are also regulated by JAs.

### 4.13. Contribution of UBL-Mediated Modifications to JA Signaling

**Figure 4:**
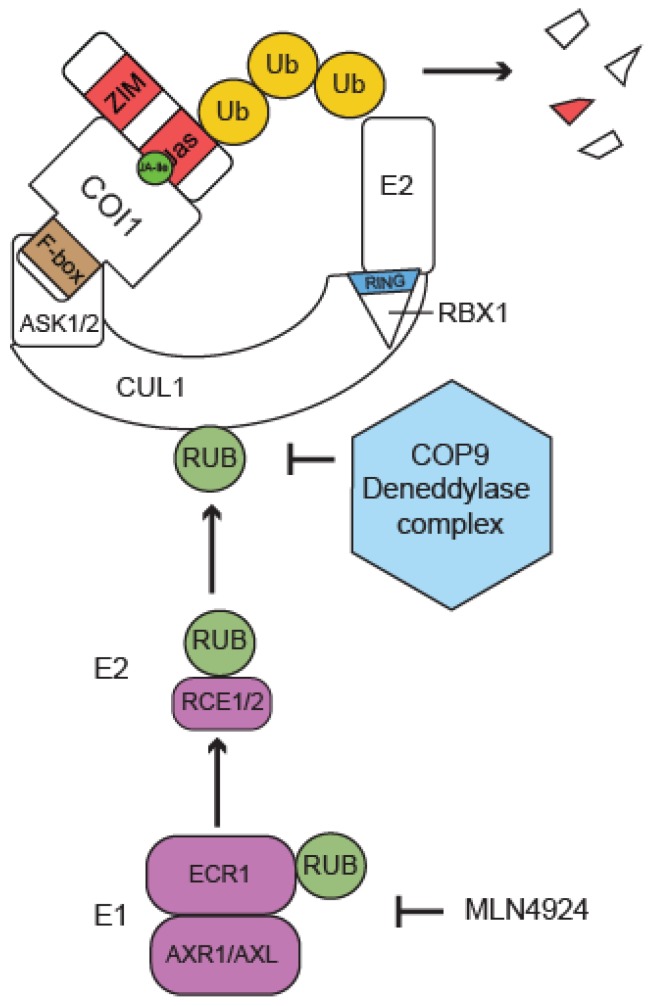
Regulation of SCF^COI1^ by Nedd8/RUB. The activity of SCF^COI1^ is modulated by cyclic attachment and removal of the ubiquitin-like modifier (UBL) Nedd8/RUB to the CUL1 subunit of the SCF complex. Accordingly, plants carrying mutations in the genes encoding components of the neddylation machinery (E1: ECR1/AXR1/AXL; E2: RCE1/2) or in the COP9 deneddylase signalosome complex are also affected in JA signaling. Defects in the RUB-conjugation pathway can also be mimicked using the E1-specific inhibitor MLN4924 [114].

As mentioned above, UBLs are small conserved proteins with high structural similarity to Ub, whose conjugation generally depends on an E1-E2-E3 cascade, as is the case for Ub conjugation [32]. Both neddylation (or RUBylation) and SUMOylation have been reported to influence JA responses in plants [40,117]. The best characterized Nedd8-conjugated proteins are the CUL components of CRL-type E3 ligases, whose activity is modulated by this modification. Components of the Nedd8-conjugation machinery are therefore required for the correct function of SCF complexes including the JA receptor component SCF^COI1^ (Figure 4) and the auxin receptor component SCF^TIR1^ [40]. Accordingly, plants deficient in Nedd8-specific E1 components (ECR1/AXR1/AXL) or E2 (RCE1/2) proteins have been reported to be JA insensitive [34,35,36,65,115]. Conversely, deneddylation of CUL components of, for example, SCF^COI1^ or SCF^TIR1^, is also essential for hormone signaling [68,129]. This is particularly well exploited by members of the Geminivirus family that inhibit JA signaling by targeting the CSN-mediated deneddylation of SCF E3 ligases [116]. Finally, the SUMO-specific E3 ligase SIZ1 (for SAP and Miz domain) has also been reported to be involved in the JA response, because *siz1-3* mutants fail to induce the expression of several JA-responsive genes under drought stress [117]. It is worth mentioning that *siz1* null mutants exhibit highly pleiotropic phenotypes. Accordingly, SIZ1 has been reported to contribute to the SUMOylation of different classes of regulatory proteins [44].

## 5. Perspectives

This review aims at summarizing the existing knowledge of the relationship between the Ub system and the JA-signaling pathway. Based on the large number of E3 ligases encoded in the model plant Arabidopsis (>1500), it is however unlikely that the E3 ligases described in this review constitute a comprehensive listing of all E3s to be involved in JA signaling. Further identification of novel E3 ligases involved in this signal transduction pathway will therefore further contribute to the expansion of our understanding of the role of the Ub system in JA signaling. In addition, improvement of the methodology for the specific identification and characterization of plant E3 ligase targets is still an issue that needs to be tackled, because very few E3 targets have been identified until now.

We have successfully used tandem affinity purification of both a single-subunit and multi-subunit E3 ligase to identify substrates [67]. By mutating the E2 RING interaction domain and potentially stabilizing the interaction of the E3 and its substrate, the discovery of substrates may be enhanced [67]. This might be more complex for multi-subunit E3 ligase complexes, because the ASK1-COI1 interaction, for example, is essential for COI1 stability [73]. However, single amino acids in TIR1 have recently been discovered to reduce the interaction of TIR1-ASK1 with the CUL1 scaffold, stabilizing the interaction with Aux/IAAs [130]. Alternatively, one may enhance substrate availability by blocking proteasomal degradation using MG132 or SCF complex functioning by MLN4924 [114].

The subsequent characterization of the E3-substrate interaction currently relies primarily on *in vitro* work, which is often not trivial. Important here is also knowledge of the E2 interacting and working together with the E3. Attempts have been made to identify plant E2-E3 pairs [18,131]. Alternative *in vivo* methods using bacteria [132] are being developed, but might not be suitable for multi-subunit E3s.

Identification of the ubiquitinated lysine(s) on target proteins would be informative, although it seems that ubiquitination in some instances can be very promiscuous [133]. As mentioned, methods to identify ubiquitination sites proteome-wide are finding their way into plant research [26], with new ones being developed [134]. Moreover, these protocols should be suitable to compare conditions such as with or without JA treatment. Such “dynamic” proteomics approaches are still lacking in the field of JA signaling, and in phytohormone signaling in general. A striking example is the fact that direct proof of the *in vivo* ubiquitination of JAZ proteins is scarce and limited to only two members of the family [67,81]. Likewise, from these studies, it was not clear whether these two JAZ proteins had been modified with a Ub or a UBL *in vivo*. Indeed, the mass spectrometry-based approaches applied in these studies led to the identification of a di-glycine modification of the respective JAZ proteins. This modification is derived from Ub or a UBL, and remains on the target protein after trypsin digestion, an intermediary step during the preparation of samples for mass spectrometric analysis. Since a covalently attached Ub- or UBL-entity on the target leaves behind the same di-glycine modification after trypsin digestion, no distinction can be made between the two.

Finally, studying the degradation dynamics of target proteins might reveal differences within protein families. With JAZ proteins, we successfully used fusions to firefly luciferase to monitor their rapid degradation upon JA perception [58,109] and, recently, an elegant sensor system was developed using the Venus fluorescent protein [135].

Taken together, progress in our knowledge of ubiquitination in plants is highly technology-driven. Hence, with the fast evolving fields of proteomics and interactomics, we expect our understanding of ubiquitination in plants to advance rapidly.
